# Mitochondrial Proteome Studies in Seeds during Germination

**DOI:** 10.3390/proteomes4020019

**Published:** 2016-06-21

**Authors:** Malgorzata Czarna, Marta Kolodziejczak, Hanna Janska

**Affiliations:** Department of Cellular Molecular Biology, Faculty of Biotechnology, University of Wroclaw, F. Joliot-Curie 14A, Wroclaw 50-383, Poland; marta.kolodziejczak@uwr.edu.pl (M.K.); hanna.janska@uwr.edu.pl (H.J.)

**Keywords:** germination, mitochondria, mitochondrial biogenesis, proteomics, post-translational modifications

## Abstract

Seed germination is considered to be one of the most critical phases in the plant life cycle, establishing the next generation of a plant species. It is an energy-demanding process that requires functioning mitochondria. One of the earliest events of seed germination is progressive development of structurally simple and metabolically quiescent promitochondria into fully active and cristae-containing mitochondria, known as mitochondrial biogenesis. This is a complex and tightly regulated process, which is accompanied by sequential and dynamic gene expression, protein synthesis, and post-translational modifications. The aim of this review is to give a comprehensive summary of seed mitochondrial proteome studies during germination of various plant model organisms. We describe different gel-based and gel-free proteomic approaches used to characterize mitochondrial proteomes of germinating seeds as well as challenges and limitations of these proteomic studies. Furthermore, the dynamic changes in the abundance of the mitochondrial proteomes of germinating seeds are illustrated, highlighting numerous mitochondrial proteins involved in respiration, tricarboxycylic acid (TCA) cycle, metabolism, import, and stress response as potentially important for seed germination. We then review seed mitochondrial protein carbonylation, phosphorylation, and *S*-nitrosylation as well as discuss the possible link between these post-translational modifications (PTMs) and the regulation of seed germination.

## 1. Introduction

**Figure 1:**
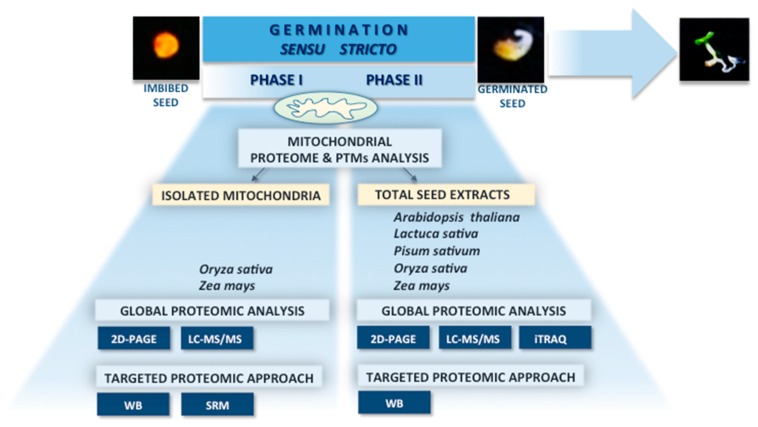
Scheme of proteomic studies of mitochondrial proteins in seeds during germination of different plant model organisms (*A. thaliana*, *L. sativa*, *P. sativum*, *O. sativa*, *Z. mays*). Mitochondrial proteome analyses of germinating seeds were performed using gel-based or gel-free approaches with either isolated organelles or the whole seeds. Targeted or global proteomic surveys were applied.

Seed germination is one of the most crucial phases in the plant life cycle and in its essence it determines the success of propagation of plant species. Germination starts with the uptake of water by a dry mature seed in a process called imbibition, and finishes with a radicle protrusion, which is a visible symptom of the completion of germination [1]. The absorption of water by a seed can be characterized as a triphasic process, starting with a rapid intake (phase I, *i.e.*, imbibition), followed by a time of limited intake (phase II, plateau phase) and eventually a period of further increase in the intake of water (phase III, postgermination phase). The first two phases describe physical and metabolic processes occurring in imbibed seeds and define germination *sensu stricto*. Soon after the germination begins, there is reactivation of seed metabolic activity, which involves repair and *de novo* synthesis of DNA, mobilization and degradation of stored proteins and mRNAs, transcription and translation of new mRNAs followed by cell elongation and protrusion of the radicle tip [2]. The third phase occurs only after germination is completed and is related to an early seedling growth [2] (Figure 1). Seed germination is an energy-demanding process that requires functioning mitochondria immediately upon imbibition. Therefore, one of the earliest events of seed germination is progressive proliferation and differentiation of mitochondria, known as mitochondrial biogenesis.

It is believed that mitochondria cannot be created *de novo*, meaning that they arise from the division of parental organelles [3]. Until now, two models of mitochondrial biogenesis have been described: the growth and division model and the maturation model [4]. The first model of mitochondrial biogenesis is based on the studies on yeast and mammalian cells [5,6]. It describes the growth and division of pre-existing mature mitochondria through a binary fission, which generally accompanies cell mitosis, while the mitochondrial mass increases during interphase [7,8]. The maturation model of mitochondrial biogenesis was initially observed in yeast and illustrates the existence of structurally and biochemically simple organelles, called promitochondria, that, by responding to specific signals, mature into fully developed and metabolically active mitochondria [9]. Numerous studies on biogenesis of mitochondria in seeds support rather the maturation model of mitochondrial biogenesis during germination [10,11,12,13]. It is proposed that biogenesis of mitochondria during germination starts from the development of structurally simple and metabolically/energetically quiescent promitochondria existing in dry mature seeds into fully active and cristae-containing organelles [4,14]. This transition is accompanied by sequential and dynamic gene expression, protein turnover, and post-translational modifications. Mobilization of any reserve transcripts and proteins as well as initiation of *de novo* protein synthesis is necessary for the proliferation of mitochondria and the completion of seed germination. The importance of protein synthesis during germination was reported earlier by Rajjou *et al.* [15] and Galland *et al.* [16] who showed using *Arabidopsis thaliana* seeds that inhibition of this process with cycloheximide arrested germination completely while inhibition of transcription with α-amanitin just delayed it.

There are several comprehensive reviews, which have been published within the last few years associated with proteome changes during seed maturation and germination [1,17,18,19,20,21]. Yet, there is relatively little information addressing seed mitochondrial proteome dynamics. In this review, we describe the current knowledge about the different proteomic approaches used to characterize the seed mitochondrial proteomes as well as limitations and challenges in proteomic studies of seed mitochondria. We also highlight the dynamics of seed mitochondrial proteomes, the relationship between transcriptome and proteome changes, and the most recent findings in terms of protein carbonylation, phosphorylation, and *S*-nitrosylation in seed mitochondria during germination of various plant model organisms.

## 2. Bioenergetics and Heterogeneity of Seed Mitochondria Structure

Early observations of mitochondrial structures with transmission electron microscopy revealed that mitochondria extracted from dry sunflower seeds were characterized by a very low density of mitochondrial matrix and discontinuous or absent outer membrane [22]. In contrast, internal membrane was continuous but with very few cristae. Interestingly, these mitochondria oxidized various respiratory substrates and produced low amounts of ATP, demonstrating that they are capable of oxidative phosphorylation (OXPHOS) [22]. Other electron microscopy studies using maize and rice embryos supported these findings [11,12]. Further studies of maize and rice seeds showed that following imbibition the undifferentiated mitochondria developed cristae, the electron density of the matrix increased and a typical mature mitochondrial structure was visible after 24 h of germination [11,12]. These structural changes were accompanied by an increase in mitochondrial import and metabolic activity as well as a rapid respiration rate [11,23]. Besides, the observations using pea, maize, and rice embryos also showed a significant increase in the rate of oxygen uptake shortly after seed imbibition, leading to the assumption that mitochondria present in dry seeds are able to synthesize a higher amount of ATP as soon as the seeds are rehydrated [12,23,24,25]. In conclusion, upon imbibition the very simple promitochondria rapidly differentiate and maturate into fully functional mitochondria.

Using sucrose density gradient centrifugations, Logan *et al.* [11] performed fractionation of crude homogenate of maize embryos prepared from dry seeds as well as from seeds, which had been germinating for different amounts of time. The obtained mitochondrial fractions from every type of the seed were composed of two distinct subpopulations: one subpopulation of a density equivalent to 22%–28% (*w*/*w*) sucrose and the other equivalent to 37%–42% (*w*/*w*) sucrose, referred to as light and heavy mitochondria, respectively. In dry seeds, both subpopulations constituted poorly developed mitochondria. However, during germination the heavy mitochondria gradually acquired typical features of fully functional mitochondria regarding structure, protein content, and metabolic activity. In contrast, the light mitochondrial subpopulation did not show any significant changes in membrane morphology, while the amount of specific proteins decreased throughout the studied germination course. It is hypothesized that the light mitochondrial subpopulation is the remainder of the mitochondria that were active during seed maturation prior to desiccation, while the heavy mitochondria are promitochondria that at the onset of imbibition develop rapidly into fully active mature mitochondria [11].

## 3. Experimental Approaches in Seed Mitochondrial Proteome Studies

Proteomics is the study of all the expressed proteins within the tissue, cell, or organelle. As a broad discipline, proteomics has been also applied to all aspects of seed biology such as seed maturation, desiccation tolerance, germination, dormancy, and vigor, using Arabidopsis as a model organism or numerous important agricultural plants (maize, rice, wheat, castor, pea, lettuce) under a variety of conditions.

Several different approaches have been applied to monitor the on-going changes in the abundance of mitochondrial proteins during seed germination: (i) a direct study of the global mitochondrial proteome variations using isolated organelles from germinating seeds [12,23]; (ii) a targeted approach to study specific mitochondrial proteins in isolated organelles from germinating seeds [11,26]; (iii) an indirect approach to describe the changes of mitochondrial proteomes in germinating seeds, using total seed protein extracts to identify and measure mitochondrial proteins [13]; (iv) an indirect study with the aim to describe the variations in total proteome in germinating seeds, in which mitochondrial proteins have been detected among many other proteins [16,27,28,29,30] (Figure 1).

To obtain an overall view of the mitochondrial proteome dynamics during germination, gel-based and gel-free comparative proteomic studies of whole germinating seeds or isolated organelles were carried out on different plant species (Figure 1; Tables S1 and S2). Gel-based methods, especially the classical two-dimensional gel electrophoresis (2D-PAGE) with post-gel identification by mass spectrometry, still dominate in seed proteomics [21]. Despite some limitations, of which the most significant is the low resolution of membrane and/or hydrophobic proteins, the 2D-PAGE approach with its variations is reasonably quantitative, and its generally high resolving power can lead to the separation of the analyzed sample into several thousand individual protein spots [31]. The obtained quantitative protein maps deliver a lot of information regarding the intactness of the protein sample, differences in abundance between two or more biological conditions, protein variants differing in molecular weight and/or pI as well as characterization of post-translational modifications, such as carbonylation, phosphorylation, glycosylation, acetylation, and methylation [31]. 2D-gel electrophoresis has been used to study changes in abundance in the total seed proteome during germination of Arabidopsis, pea, rice, and lettuce seeds [27,28,29,30] (Table S1). In the studies performed by Howell *et al.* [12,23] two-dimensional separation of mitochondrial proteins isolated from rice embryos was applied to examine the effects of oxygen on mitochondrial biogenesis during rice germination (Table S1). An interesting gel-based approach was used by Galland *et al.* [16] who combined two-dimensional gel electrophoresis with radiolabeled proteomics using a radiolabeled [^35^*S*]-methionine, to study *de novo* protein synthesis and stability during Arabidopsis seed germination (Table S1).

With the rapid development of mass spectrometry (MS) technology, the global analysis of protein composition, quantity and post-translational modifications using high-resolution mass spectrometry has been successfully applied in cellular and organellar proteomics, including seeds (Tables S1 and S2). In past years most of the proteomic studies relied on tandem mass spectrometry (MS/MS) with protein samples digested into peptides, separated by liquid chromatography (LC), ionized, and analyzed by the mass spectrometer [32]. In this MS-based approach called shotgun proteomics, the vast number of detected fragment ion spectra is used to identify and quantify the particular peptide in a sample as well as to locate modified amino acid residues. However, the random peptide selection process used in shotgun proteomics leaves more abundant peptides more likely to be selected for fragmentation and therefore analysis, resulting in insufficient identification of less abundant proteins. The shotgun mass spectrometry strategy was applied by Law *et al.* [13] (Table S1) to quantify the abundance of total proteins of Arabidopsis at different time points during seed germination and to compare the observed protein changes with the corresponding transcript level.

In the last decade, protein quantification through incorporation of stable isotopes to the studied samples has become the most frequently used MS-based proteomic strategy. Among different quantitative methods, which rely on the stable isotope labeling, chemical modification of the tryptic peptides with isobaric tags for relative and absolute quantitation (iTRAQ) has become a popular tool in quantitative cellular and organellar proteomics, including plant mitochondria [33]. iTRAQ offers the possibility to compare the quantity of up to eight protein samples in the same experiment, which allows analysis of time-course studies [34]. More recently, Han *et al.* [29] applied iTRAQ in combination with a 2D gel-based approach to perform a systematic quantitative proteomic analysis of rice embryos dissected from germinating at different time point seeds (Table S1).

In the past few years, Selected Reaction Monitoring (SRM), known also as Multiple Reaction Monitoring (MRM), has emerged as the novel targeted proteomic approach. This gel-free, mass spectrometry-based technique is used for absolute quantification of a protein target or group of proteins from a variety of sources, including plant seeds. The SRM assay is able to quantify a predefined protein with extreme sensitivity, matching highly sensitive and specific immunological assays such as Western Blot or ELISA [35], and reliably distinguish between similar isoforms of proteins where traditional antibodies are insufficient [36,37]. Yet, given that it is a targeted approach, a prior knowledge of the protein of interest in the sample is obligatory and a relatively small number of protein targets (up to 100) can be examined in one SRM workflow. Lately, SRM has been successfully applied in diverse plant mitochondria proteomic studies [26,37,38]. Using this technique, the abundance of the basic amino acid carrier involved in arginine metabolism in rice seed mitochondria during germination under aerobic and anaerobic conditions has been quantified [26] (Table S1). Additionally, the SRM approach has been implemented to examine the changes in abundance of protein targets belonging to the OXPHOS components during *A. thaliana* germination course [39].

While the general identification of mitochondrial proteins in seeds was performed using both gel-based (2D-PAGE) and MS-based proteomic approaches (LC-MS/MS, iTRAQ, SRM) (Table S1), the identification of PTMs (such as carbonylation and phosphorylation) of mitochondrial proteins in dry and in germinating seeds has been dominated by in-depth MS-based studies [40,41,42,43] (Table S2). Zhang *et al.* [40] utilized biotin hydrazide labeled chromatography, which allows enrichment of carbonylated proteins, combined with the sequential window acquisition of all theoretical fragment ion spectra (SWATH) method to analyze the protein carbonylation pattern in rice embryos isolated from different stages of seed germination (Table S2). To identify phosphorylated proteins in germinating seeds (rice and maize), different phosphopeptide enrichment methods, such as strong-cation exchange (SCX) or polymer-based metal ion affinity capture (PolyMAC) were implemented, followed by analyses using either shotgun approach and LC-MS/MS, or nano-liquid chromatography coupled with tandem mass spectrometry (nano-LC-MS) [41,42,43] (Table S2).

## 4. Limitations in Seed Mitochondrial Proteome Studies

Intact and pure mitochondria are fundamental for the measurements of their activity and any other assays, including proteomic analysis. Developing and germinating seeds are considered a difficult material for the isolation of mitochondria because of their high density and compactness [44]. Additionally, low water content in seeds requires high-pressure forces during tissue grinding, which might lead to the partial disruption of the organelles and reduction of the quality of the isolated mitochondria. Several studies on isolation of mitochondria from dry seeds resulted in obtaining highly damaged mitochondria [45,46,47]. For most protocols for mitochondria isolation used in any proteomic studies it has been thus more beneficial to utilize germinating seeds since mitochondrial integrity and functionality improves during germination while the higher water content allows for easier organelle isolation, yielding better quality mitochondria. In addition to that, analyses of seed mitochondrial proteins obtained from the isolated organelles have been mostly performed on large-sized seeds such as maize or rice [11,12,23,26] (Figure 1). Yet, a recent study by Ahmed and Fu [48] resulted in an improved protocol for the isolation of mitochondria from dry as well as small-sized seeds (*i.e.*, Arabidopsis). However, this protocol is originally applied to the studies on mitochondrial DNA and has not yet been tested on isolated whole mitochondria subjected to proteomic surveys. Also, it might bear further difficulties due to the large amount of small-sized seeds needed to yield sufficient amounts of mitochondria.

Apart from the difficulties of obtaining pure and intact mitochondria from the seed, further obstacles can arise because of the high abundance of seed storage proteins (SSPs). Storage proteins are one of the major reserves (apart from starch and lipid) and the most abundant proteins in seeds. The plethora of seed storage proteins might be a great benefit when one studies SSP; however, this high abundance of storage proteins can interfere with total and organellar proteome studies, especially those employing gel-based approaches, in which lower-abundant proteins can be masked [18]. Therefore, it might be advisable to remove SSP during protein extraction in order to make lower-abundance proteins detectable. Miernyk and Hajduk [18] provided a short overview of various possible strategies to reduce storage proteins in a given seed protein sample. Recently, a removal of storage proteins by polyethylene glycol fractionation was utilized successfully in lettuce seeds, improving the detection of less-abundant proteins on 2D-PAGE gels [30].

## 5. The Changes in Abundance of Mitochondrial Proteins during the Germination Course

The dynamics of mitochondrial protein abundance in germinating seeds has been observed in many plant species, such as *Oryza sativa* (rice) [12,23,26,29], *Pisum sativum* (pea) [28], *Arabidopsis thaliana* [13,16,27,49], *Lactuca sativa* (lettuce) [30], and *Zea mays* (maize) [11]. The list in Table S1 highlights seed mitochondrial proteins, which have shown changes in abundance at different stages during the course of germination, and have been either identified in global proteomic analyses (2DE, MS, iTRAQ) or examined using the targeted proteomic approach (WB, SRM) in different plants. We grouped the examined proteins into several functional categories (Table S1). Most mitochondrial proteins belonged to the six major following categories: “Metabolism”, “Respiration”, “Tricarboxylic acid cycle (TCA)/Carbon metabolism”, “Import/Transport”, “Stress response”, and “Chaperones and proteolytic system”. Among the studied mitochondrial proteins, several enzymes involved in metabolism (aldehyde dehydrogenase, monodehydroascorbate reductase, glyceraldehyde-3-phosphate dehydrogenase), respiration (Rieske protein, cytochrome *c*, the alpha and beta subunits of ATP synthase), TCA and carbon metabolism (the alpha and beta subunits of pyruvate dehydrogenase E1, citrate synthase, malate dehydrogenase, phosphoenolpyruvate carboxykinase), import/transport (Tom40, voltage-dependent anion channel (VDAC), adenine nucleotide translocator), stress response (manganese superoxide dismutase) and development (late embryogenesis abundant protein) as well as chaperones and proteins of the proteolytic system (Hsp60, Hsp70, mitochondrial processing peptidase) appeared in more than one experimental set-up and different studied plant species (Table S1). The overrepresentation of these proteins may indicate their high abundance in seed mitochondria and underlines their importance in the process of seed germination.

Galland *et al.* [16] performed an interesting study, which provided a detailed description of the dynamics of the Arabidopsis total seed proteome at different time points during the germination course. In this 2D gel-based survey not only differential protein abundance but also *de novo* protein synthesis was examined. The germination assay was carried out for the time of 48 h that corresponds to the three major phases of seed water uptake [50]. Here we focus on the proteomic changes occurring in the Arabidopsis seed mitochondria from 0 to 24 h of germination time, which refers to the first two phases of water uptake and defines germination *sensu stricto* [16] (Figure 1). Among the identified 475 protein spots corresponding to 257 non-redundant proteins, there were mitochondrial proteins that either differentially accumulated (up or down) or remained constant during the germination course. Some of these identified mitochondrial proteins were radioactively labeled, and therefore *de novo* synthesized (referred to as neosynthesized) (Table S1). These neosynthesized mitochondrial proteins showed in most cases increased abundance during germination, such as glutamate dehydrogenase 1 or 3, monodehydroascorbate reductase, glyceraldehyde-3-phosphate dehydrogenase, succinate-semialdehyde dehydrogenase, the subunit beta of ATP synthase, aconitate hydratase 3, phosphoenolpyruvate carboxykinase, the subunit beta of mitochondrial processing peptidase, Hsp60, and translation elongation factor EF-Tu. However, there were also mitochondrial proteins identified that, although being neosynthesized, displayed constant (succinyl-CoA ligase alpha-chain, the flavoprotein subunit of succinate dehydrogenase, and Hsp70-2) or decreased abundance (the alpha subunit of ATP synthase, superoxide dismutase 2) within the time of 24 h germination (Table S1). Furthermore, between the detected mitochondrial proteins there were several proteins that were not radioactively labeled. Interestingly, some of them (glutamate dehydrogenase 2, dihydrolipoamide dehydrogenase 2 or 1, citrate synthase, and malate dehydrogenase) showed increased abundance in spite of the fact that they were not *de novo* synthesized. This finding demonstrates the importance of post-translational regulation of seed mitochondrial proteins during the germination process. The other not neosynthesized proteins displayed either decreased abundance (NADH-ubiquinone oxidoreductase 75 kDa subunit, superoxide dismutase 1, and late embryogenesis abundant protein) or were constant during the studied germination phases (glutamate dehydrogenase, succinyl-CoA ligase beta-chain, and formate dehydrogenase) (Table S1).

Howell *et al.* [12,23] performed an important study to examine the effects of oxygen on mitochondrial biogenesis during rice embryo germination. While the mitochondrial morphology appeared to be independent of oxygen availability, the comparisons of abundance of mitochondrial proteins obtained from embryos germinating under aerobic and anaerobic conditions pointed out some differences in abundance in response to an oxygen signal [12,23] (Table S1). Several proteins involved in metabolism and TCA cycle (mitochondrial aldehyde dehydrogenase 2a, the alpha and beta subunits of pyruvate dehydrogenase E1, pyruvate dehydrogenase/2-oxo-glutarate dehydrogenase complex, E2 component, the beta chain of succinyl-CoA ligase), respiratory chain (cytochrome *c*, Rieske protein (RISP), Cox2) as well as Hsp70 and mitochondrial elongation factor Tu, were lower in abundance in anaerobic mitochondria [23]. Similarly, both subunits of the mitochondrial processing peptidase (MPP), which in plants is integrated into the cytochrome *bc_1_* complex, were detected in lower amount under anaerobic conditions, consistently with decreased abundance of RISP [23]. Notably, although the components of the protein import apparatus (TIM17/TIM22/TIM23 family proteins and Tom20) were several times more abundant [23] (Table S1), the general capacity of the mitochondrial import pathway was significantly lower in anaerobically germinating embryos. These results indicated that a lack of oxygen suppresses the normal increase in mitochondrial protein import observed during germination under aerobic conditions [12]. The presence of oxygen leads to both increase in import capacity and abundance of the cytochrome *bc1* complex suggesting a link between the mitochondrial protein import apparatus and the respiratory chain [23].

Two isoforms of mitochondrial aldehyde dehydrogenase, ALDH2a and ALDH2b, from germinating rice embryos showed an interesting opposite regulation at the level of protein abundance in response to the low-oxygen signals [23] (Table S1). An involvement of the ALDH2 isoforms in rice seedlings during re-aeration after submergence has been discussed previously by Tsuji *et al.* [51], who suggested an essential role of ALDH2 in the detoxification of acetaldehyde in low-oxygen stress in plants.

A late embryogenesis abundant (LEA) protein was found to be higher in abundance in rice mitochondria isolated from anaerobically germinating embryos [23], in comparison to the seeds growing in the presence of oxygen [12,29] (Table S1). Earlier, a role of mitochondrial LEA protein in protecting stored mitochondrial proteins during desiccation in pea seeds was observed [52]. The up-regulation of LEA in anaerobically germinating rice seeds suggests that this protein protects mitochondria in response to the deficit of oxygen as well.

Two mitochondrial proteins related to reactive oxygen species (ROS) detoxification, namely manganese superoxide dismutase (MSD, identified in several experimental approaches and different plant species) and catalase 3 were found to change in abundance during germination (Table S1). While catalase 3 has been shown to increase in abundance in two different experimental set-ups, we found an opposite behavior of superoxide dismutase, being up and down regulated. However, the observed discrepancies among the MSD abundances appear to be due to the differences in the studied plant species and/or germination conditions [12,16,28,29,30]. It has been suggested that successful germination arises from the activity of ROS molecules that function in a certain oxidative window as signaling molecules without harmful consequences for the cell [53]. Interestingly, Galland *et al.* [16] detected one of the manganese superoxide dismutase isoforms (At3g65350) as the neosynthesized protein, which displayed maximum neosynthesis between 16 and 24 h of germination. These results may indicate that the synthesis of antioxidants starts after approximately 16 h of germination in at least Arabidopsis seeds, while the “oxidative window for germination” [53] would appear earlier, at the beginning of the germination program.

## 6. Relationship between Proteomic and Transcriptomic Changes in Seeds during Germination

Changes in the abundance of mitochondrial proteins in seeds during the germination course may result from the differential expression of the transcripts that encode them. Previous studies revealed that in dry seeds there is a large number of long-lived transcripts (between 12,000 and 17,000) representing stored mRNA species, which survive the desiccation process and are thought to play a central role in early stages of germination [54,55,56,57,58]. Interestingly, a major pool of the stored mRNAs constitutes transcripts encoding LEA and seed storage proteins, reflecting the processes of seed maturation and preparation for the subsequent germination [59]. High accumulation of LEA has been observed at transcript and protein levels in mitochondria of dry mature pea seeds [52]. Kimura and Nambara [57] evidenced that dry seeds contain all the components of the transcriptional and translational machineries, which are quickly activated at the onset of imbibition when rapid metabolic changes occur. It has been acknowledged that in seeds, protein synthesis occurs from the long-lived stored mRNA species as well as from the transcripts that have been synthesized by *de novo* transcription during the early stages of germination [15]. Recent proteomic studies additionally highlighted the dynamics of selective mRNA translation in seeds during the germination time course [16].

Several studies on mitochondrial biogenesis during seed germination revealed the presence of transcriptional and post-transcriptional regulation of this process. The quantitative RT-PCR assays of genes encoding mitochondrial components in rice embryos revealed the presence of a sequential order of transcription over the 48 h germination course [12]. The analyses revealed that at the very start of germination (1–3 h of imbibition), transcripts encoding the mitochondrial protein import apparatus increased rapidly in abundance, followed by the genes encoding proteins associated with mitochondrial transcription, translation, and division. Progressive increase in expression with a peak developing after approximately 12–24 h of germination showed transcripts encoding components of the TCA cycle and respiratory chain. These increases in gene expression associated with TCA and the electron transport chain correlated with a strong increase in protein abundance observed from Western Blot and 2D-gel proteomic analyses [12] (Table S1). On the contrary, the components of the mitochondrial import machinery showed the highest protein abundance in dry rice embryos and declined markedly during germination, even though their transcript level was relatively stable. This lack of transcript/protein correlation presumably indicates an active degradation of the protein components of the import machinery during rice embryo germination.

Using the transcript abundance changes from the previously published microarray data [58], Law *et al.* [13] performed in-depth analyses of the genes encoding mitochondrial components to gain a detailed insight into the molecular aspects of mitochondrial biogenesis during Arabidopsis seed germination. Upon examination of the expression profiles during cold stratification and at different time points of germination a model describing the sequence of transcriptomic events was established. First, at the end of stratification the transient expression of genes encoding proteins associated with DNA and RNA metabolism was observed. Second, early in germination, the peak in expression of genes encoding proteins associated with protein synthesis and import occurred. This was followed by the progressive increase in transient expression of genes encoding electron transport chain components, showing maximal expression between 24 and 48 h of germination [4,13]. Furthermore, using quantitative information for 178 total seed proteins obtained by shotgun LC-MS/MS and the expression profile of the corresponding transcripts, a heat map of transcript and protein abundance grouped into functional units was constructed. Significant positive correlations were revealed for 81 out of the 178 transcripts/proteins and only 15 showed significant negative correlations. Among the 178 identified seed proteins, 14 mitochondrial proteins were found of which nine showed a significant positive correlation. Most of these mitochondrial proteins constitute metabolic and transport components and showed increased abundance during the time of germination [13] (Table S1). A positive correlation between transcript and protein abundance was additionally shown for several transcripts encoding the components of the electron transport chain. Using quantitative Western Blot data the highest accumulation of these proteins was observed at the end of the germination course. Similarly to germinating rice embryos [12], no transcript/protein abundance correlation for the components of the mitochondrial import apparatus was observed.

Overall, there is coordinated dynamics of expression for the majority of the studied mitochondrial transcripts and proteins during seed germination.

## 7. Post-Translational Modifications of Mitochondrial Proteins in Seeds

Earlier proteomic investigations emphasized the pivotal role of post-translational control in seed germination [15,57]. It is known that seed proteins are subjected to a broad type of PTMs and proteolysis, which may affect protein function, localization, and stability. However, there is insufficient information on the type of post-translational modifications of mitochondrial proteins in seeds and the possible link between these PTMs and seed germination.

### 7.1. Carbonylation of Seed Mitochondrial Proteins

Carbonylation is an irreversible type of protein oxidation that can be induced by ROS, reactive nitrogen species (RNS) or by-products of oxidative stress [60]. The addition of carbonyl groups to the protein may result in loss of its function and degradation or formation of toxic to the cell protein aggregates. Carbonylated proteins have been identified throughout the entire plant life cycle. Numerous studies indicated that selective carbonylation of plant specific proteins may constitute a molecular mechanism involved in different physiological processes [61,62,63,64,65]. It has been reported that germination of Arabidopsis seeds is triggered by changes in the carbonylation level of seed storage proteins [61], while the study on sunflower seeds has shown the role of differential protein carbonylation in dormancy alleviation [63]. Although protein carbonyl groups have been found in all plant cellular compartments [61,62,64,66,67], some studies indicate that it is mitochondria that house the highest concentration of carbonylated proteins [66,68]. Recently, Smakowska *et al.* [69] thoroughly reviewed developmentally dependent pattern of protein carbonylation in plants with emphasis on mitochondrial proteomes.

Here, we present a list of carbonylated proteins identified so far in Arabidopsis and rice embryo mitochondria with the 2,4-dinitrophenylhydrazine (DNPH) immunoassay [61] and biotin hydrazide labeling-affinity chromatography combined with the SWATH quantitative method [40] (Table S2). The latter approach, applied for the first time in plants, provided more comprehensive analysis on the dynamics of protein carbonylation during seed germination and let to the identification of more and new carbonylated proteins than previous 2-DE based studies [60,63]. The carbonylated mitochondrial proteins are implicated in different cellular responses and processes such as stress response, redox homeostasis, chaperones, tricarboxylic acid cycle, respiration, and metabolism, and they showed in most cases increased carbonylation level during germination. One of the most interesting findings is that some chaperones (Hsp60, Hsp70) and antioxidants (MSD and peroxiredoxin, Prx)—proteins related with defense against oxidative stress—are themselves sensitive to this oxidative modification during germination (Table S2). Despite the antioxidant functions of MSD and Prx [70,71] and the protective role of chaperones, which probably act as a shield protecting other proteins against oxidative damage [72], it seems that these proteins are relatively susceptible to carbonylation and under prolonged oxidative stress they could become dysfunctional [69]. Among the detected carbonylated proteins, the TCA cycle and OXPHOS enzymes, such as aconitate hydratase 2 (ACO2), isocitrate dehydrogenase, succinate dehydrogenase, as well as the beta subunit of ATP synthase were found in Arabidopsis and rice seeds [40,61]. These proteins might be rapidly inactivated during oxidative stress mainly because of their location in proximity of the ROS-generating sites [73].

Seed protein carbonylation is a type of protein oxidation that attracts growing attention, however, the role of carbonylated mitochondrial proteins in germinating seeds is still elusive. The presence of this PTM in seed mitochondria might be simply the result of an enhanced oxidative stress, which occurs during germination, but it might also have a physiological meaning and could be beneficial for seed germination. Further studies are required to confirm both hypotheses.

### 7.2. Phosphorylation of Seed Mitochondrial Proteins

Reversible protein phosphorylation is one of the most well-studied and important post-translational modifications. However, despite the large variety of phosphoproteomic studies on plants, the involvement of protein phosphorylation in seed physiology is still rather poorly documented. Yet, *de novo* protein phosphorylation has been shown to occur during Arabidopsis seed germination [74]. Previously, the presence of phosphorylated LEA proteins and seed storage proteins (12S cruciferins) in Arabidopsis dry seeds was shown [75,76]. It was suggested that the phosphorylation of SSPs could be a form of phosphorus storage until germination begins. Additionally, several proteins involved in the process of translation were differentially phosphorylated during wheat and maize seed germination [77,78], indicating that during germination protein translation is regulated by phosphorylation.

In plants, phosphorylation of mitochondrial proteins has been thoroughly examined (for reviews see [79,80]). In this work we put together phosphorylated mitochondrial proteins, which have been found so far in germinating seeds [41,42,43] (Table S2). Notably, only six seed mitochondrial phosphoproteins have been identified. Taking into account 64 phosphorylated mitochondrial proteins reported in plants to date [80], this amount is astonishingly low. One of the phosphorylated mitochondrial proteins observed in maize embryos is a small heat shock protein, HSP22 [41] (Table S2). Earlier, this protein was shown to accumulate in pea seed mitochondria [81]. The high abundance and apparent regulation by phosphorylation implies an important role of HSP22 during seed germination. Other phosphorylated mitochondrial proteins identified in seeds are involved in energy metabolism (pyruvate dehydrogenase E1 component, subunit alpha; cytochrome *c* biogenesis FN protein, and alternative oxidase 3) [41,42] and protein translation (60S ribosomal protein L5 and ribosomal protein L18) [43] (Table S2). The regulation of mitochondrial protein synthesis by protein phosphorylation has been also observed in mammalian mitochondria [82]. Identification of phosphorylated cytochrome *c* biogenesis FN protein indicates that during seed germination the maturation pathway of *c*-type cytochromes is likely regulated by reversible phosphorylation [41]. Further in-depth research on seed sub-cellular phosphoproteomics might be helpful to understand the mechanism of reversible protein phosphorylation controlling mitochondrial biogenesis and seed germination.

### 7.3. S-Nitrosylation of Seed Mitochondrial Proteins

*S*-Nitrosylation is a reversible covalent protein modification, which results from the attachment of a nitric oxide (NO) moiety to the thiol side chain of a cysteine residue. This PTM is believed to impact protein conformation, function, and/or location. The biotin switch technique (BST) or related approaches have been used to identify the *S*-nitrosylated proteins in all plant cellular compartments, indicating the extent and importance of regulatory mechanism by which NO modulates protein functions and cell signaling in the plant life cycle [83,84,85,86,87,88,89,90,91,92,93]. Several studies have shown that NO affects seed dormancy, germination, and sensitivity of seeds towards abscisic acid (ABA) [94,95]. Recent findings demonstrated that in Arabidopsis *S*-nitrosylation of transcription factor ABI5 facilitates its degradation and promotes seed germination [96]. Despite the apparent involvement of NO in promoting seed germination, most of the NO direct protein targets remain unknown.

The importance of NO-mediated regulation of metabolism in mitochondria of germinating seeds has been pointed out by hypoxia-related studies. Due to the restricted permeability of the outer layers, seeds could experience limited oxygen supply that may lead to the reduction of mitochondrial respiration and synthesis of ATP [97]. It is proposed that to avoid the risk of anoxia, seeds developed the regulatory mechanism of low-oxygen sensing via NO-mediated inhibition of cytochrome *c* oxidase (COX), which in consequence blocks further oxygen consumption. This inhibition of COX activity results from the binding of NO to the heme a3/copper B binuclear center of cytochrome *c* oxidase [98]. However, the biological effects of NO on seed mitochondrial proteins could also be mediated through chemical modifications, such as *S*-nitrosylation. Surprisingly, according to our knowledge, there is only one mitochondrial protein, a beta subunit of the ATP synthase, which was found to be *S*-nitrosylated in seed mitochondria [74] (Table S2). It is assumed that the *S*-nitrosylated ATP synthase is inactive since a homologous protein in rat fatty liver appeared to be inhibited by this PTM [99]. In order to verify this assumption further more detailed experiments are required.

## 8. General Outlook and Challenges in Seed Proteomic Studies

The proteomic studies discussed in this review have summarized the dynamics of mitochondrial proteomes and revealed numerous mitochondrial proteins that are potentially important for seed germination of different plant species. Yet, a direct comparison of the changes in abundance of a particular protein in one functional group even within the same plant species might not be always accurate due to the following reasons: (i) the type of the studied sample (isolated mitochondria or whole seeds); (ii) different stages and time of germination, preceded or not by a cold stratification treatment; (iii) different proteomic assays—gel-based or gel-free.

The success of sub-cellular proteomics lies undoubtedly in the high quality of the protein sample. Obtaining pure and intact organelles is critical and determines the extent of the proteome coverage and detection of low abundant proteins. To overcome the limitations in isolation of pure organellar fractions for studies of seed mitochondrial proteins, further technical advancements are of great importance in quantitative proteomic surveys. Isolating mitochondria from seeds, especially from small-sized seeds, is a challenging task and will probably continue to remain difficult, thus complicating mitochondrial proteomic assays. For seeds, from which the isolation of organelles is practically impossible, implementation of targeted proteomic approaches, such as SRM, may provide invaluable information about the dynamics of a specific group of mitochondrial proteins during different stages of seed germination. Additionally, a combination of targeted and global quantitative proteomic approaches will likely expand the knowledge about seed mitochondria and reveal novel aspects of mitochondrial biogenesis during seed germination. Furthermore, more comprehensive high throughput research is needed to gain information about the type and extent of post-translational modifications of individual proteins in seed mitochondria. The identification of PTMs should rely not only on the type of modification, but it should also determine the quantity of the particular PTM relative to the abundance of the modified protein. Because of the specificity towards protein targets, phosphorylation, *S*-nitrosylation and carbonylation of seed mitochondrial proteins might regulate their activity, turnover or interactions and, in consequence, metabolic and energetic processes involved in seeds during germination. Whether these mitochondrial PTMs represent a common pathway of regulation of seed germination is still an open question.

Recent development of matrix-assisted laser desorption/ionization (MALDI)-imaging mass spectrometry (MSI) and its utilization in plant tissue, including seeds, to analyze proteins, peptides, lipids, and various metabolites is an emerging and promising analytical tool for spatial distribution of different kinds of molecules and their relative abundance [100,101]. Furthermore, an integration of post-genome methodologies, such as transcriptomics, proteomics, metabolomics, lipidomics, and interactomics will likely provide a more comprehensive insight into the seed physiology and the role of mitochondria in the germination process.

## Supplementary Materials

The following are available online at http://www.mdpi.com/2227-7382/4/2/19/s1. Table S1: Overview of the dynamics of mitochondrial proteins identified in germinating seeds using gel-based and gel-free proteomic approaches in different plant species, Table S2: Overview of carbonylated, phosphorylated and *S*-nitrosylated mitochondrial proteins identified in germinating seeds using gel-based and gel-free proteomic approaches in different plant species.

## Abbreviations

The following abbreviations are used in this manuscript:

MS	mass spectrometry
2D-PAGE	two-dimensional polyacrylamide gel electrophoresis
SRM	selected reaction monitoring
PTMs	post-translational modifications
SSPs	seed storage proteins
ROS	reactive oxygen species
RNS	reactive nitrogen species
NO	nitric oxide
