# Supplementary material for: Mitochondrial Proteome Studies in Seeds during Germination
Source: Proteomes. 2016 Jun 21;4(2):19. doi: 10.3390/proteomes4020019 (PMC5217346; doi:10.3390/proteomes4020019)
Supplement: Supplementary file 1 [file proteomes-04-00019-s001.pdf]

Supplementary Materials: Mitochondrial Proteome Studies in Seeds during Germination

Malgorzata Czarna, Marta Kolodziejczak and Hanna Janska

Table S1. Overview of the dynamics of mitochondrial proteins identified in germinating seeds using gel-based and gel-free proteomic approaches in different plant species.

Gene Locus/Accession Number	Name of Protein	Change in Abundance during Germination	Method	Studied Sample	Species/References
		**Metabolism**			
Os02g49720	Aldehyde dehydrogenase	Increased	iTRAQ	Whole seed	*O. sativa*/Han *et al.*, 2014 [1]
Os02g49720	Mitochondrial aldehyde dehydrogenase ALDH2a	Increased under aerobic conditions	2DE	Mitochondria	*O. sativa*/Howell *et al.*, 2006 [2]
Os02g49720	Mitochondrial aldehyde dehydrogenase ALDH2a	Decreased under anaerobic conditions	2DE	Mitochondria	*O. sativa*/Howell *et al.*, 2007 [3]
Os06g15990	Aldehyde dehydrogenase ALDH2b	Decreased under aerobic conditions	2DE	Mitochondria	*O. sativa*/Howell *et al.*, 2006 [2]
Os06g15990	Aldehyde dehydrogenase ALDH2b	Increased under anaerobic conditions	2DE	Mitochondria	*O. sativa*/Howell *et al.*, 2007 [3]
AAL77005	Aldehyde dehydrogenase, partial	Increased	2DE	Whole seed	*P. sativum*/Wang *et al.*, 2012 [4]
CAD70620	Branched-chain amino acid aminotransferase-like protein	Increased	2DE	Whole seed	*P. sativum*/Wang *et al.*, 2012 [4]
ABZ10818	Ornithine aminotransferase	Increased	2DE	Whole seed	*P. sativum*/Wang *et al.*, 2012 [4]
XP_002509611	Aminomethyltransferase, putative, similar to *A. thaliana* mitochondrial-type protein COG0354	Decreased	2DE	Whole seed	*P. sativum*/Wang *et al.*, 2012 [4]
At2g30970	Aspartate aminotransferase 1	Increased	MS	Whole seed	*A. thaliana*/Law *et al.*, 2012 [5]
At5g18170 or At3g03910	Glutamate dehydrogenase 1 or 3	Increased and neosynthesized	2DE	Whole seed	*A. thaliana*/Galland *et al.*, 2014 [6]
At5g18170	Glutamate dehydrogenase 1	Constant and NOT neosynthesized	2DE	Whole seed	*A. thaliana*/Galland *et al.*, 2014 [6]
At5g07440	Glutamate dehydrogenase 2	Increased and NOT neosynthesized	2DE	Whole seed	*A. thaliana*/Galland *et al.*, 2014 [6]
At4g37930	Serine hydroxymethyltransferase 1 (SHM1)	Increased	2DE	Whole seed	*A. thaliana*/Fu *et al.*, 2005 [7]

At5g26780	Serine hydroxymethyltransferase 2 (SHM2)	Increased	2DE	Whole seed	*A. thaliana*/Fu *et al.*, 2005 [7]
Os04g01590	Arginase	Increased under anaerobic and decreased under aerobic conditions	WB	Mitochondria	*O. sativa*/Taylor *et al.*, 2010 [8]
At1g63940	Monodehydroascorbate reductase, putative	Increased	MS	Whole seed	*A. thaliana*/Law *et al.*, 2012 [5]
At1g63940	Monodehydroascorbate reductase	Increased and neosynthesized	2DE	Whole seed	*A. thaliana*/Galland *et al.*, 2014 [6]
At1g63940	Monodehydroascorbate reductase	Decreased	2DE	Whole seed	*A. thaliana*/Fu *et al.*, 2005 [7]
At3g04120	Glyceraldehyde-3-phosphate dehydrogenase, C subunit	Increased	MS	Whole seed	*A. thaliana*/Law *et al.*, 2012 [5]
At3g04120 or At1g13440	Glyceraldehyde-3-phosphate dehydrogenase, C subunit	Increased and neosynthesized	2DE	Whole seed	*A. thaliana*/Galland *et al.*, 2014 [6]
Q6RUQ2	Glyceraldehyde-3-phosphate dehydrogenase	Decreased	2DE	Whole seed	*L. sativa*/Wang *et al.*, 2015 [9]
At3g58610	Ketol-acid reductoisomerase	Increased	MS	Whole seed	*A. thaliana*/Law *et al.*, 2012 [5]
D6QSY0	Ketol-acid reductoisomerase	Increased	2DE	Whole seed	*L. sativa*/Wang *et al.*, 2015 [9]
At1g79440	Succinate-semialdehyde dehydrogenase (SSADH1)	Increased and neosynthesized	2DE	Whole seed	*A. thaliana*/Galland *et al.*, 2014 [6]
Os10g40540	Putative enoyl-CoA-hydratase	Increased under aerobic conditions	2DE	Mitochondria	*O. sativa*/Howell *et al.*, 2006 [2]
Os03g52690	Unknown protein (homology to inosine-5′-monophosphatase dehydrogenase)	Increased under aerobic conditions	2DE	Mitochondria	*O. sativa*/Howell *et al.*, 2006 [2]
		**Respiration**			
AtMg00070	Nad9	Increased	WB	Whole seed	*A. thaliana*/Law *et al.*, 2012 [5]
At5g67590	Ndufs4	Increased	WB	Whole seed	*A. thaliana*/Law *et al.*, 2012 [5]
At5g37510	NADH-ubiquinone oxidoreductase 75 kDa subunit, mitochondrial [Precursor]	Decreased and NOT neosynthesized	2DE	Whole seed	*A. thaliana*/Galland *et al.*, 2014 [6]
At5g13440	Rieske protein (RISP)	Increased	WB	Whole seed	*A. thaliana*/Law *et al.*, 2012 [5]
Q6H4M9	Rieske protein (RISP)	Increased under aerobic conditions	WB	Mitochondria	*O. sativa*/Howell *et al.*, 2006 [2]; *O. sativa*/Howell *et al.*, 2007 [3]
Q6H4M9	Rieske protein (RISP)	Slightly increased under anaerobic conditions	WB	Mitochondria	*O. sativa*/Howell *et al.*, 2007 [3]

At1g22840	Cyt c	Increased	WB	Whole seed	*A. thaliana*/Law *et al.*, 2012 [5]
Q0DI31	Cyt c	Increased under aerobic conditions	WB	Mitochondria	*O. sativa*/Howell *et al.*, 2006 [2]; *O. sativa*/Howell *et al.*, 2007 [3]
Q0DI31	Cyt c	Low and constant under anaerobic conditions	WB	Mitochondria	*O. sativa*/Howell *et al.*, 2007 [3]
Osm1g00330	Cox2	Increased under aerobic and constant under anaerobic conditions	WB	Mitochondria	*O. sativa*/Howell *et al.*, 2007 [3]
P05493	ATP synthase subunit alpha, mitochondrial	Decreased	2DE	Whole seed	*P. sativum*/Wang *et al.*, 2012 [4]
AtMg01190	ATP synthase subunit alpha, mitochondrial	Decreased and neosynthesized	2DE	Whole seed	*A. thaliana*/Galland *et al.*, 2014 [6]
P05494	ATP synthase subunit alpha, mitochondrial	Constant	WB	Mitochondria	*Z. mays*/Logan *et al.*, 2001 [10]
P15998	ATP synthase, alpha subunit	Increased under aerobic conditions/Constant under aerobic conditions	2DE/WB	Mitochondria	*O. sativa*/Howell *et al.*, 2006 [2]; *O. sativa*/Howell *et al.*, 2007 [3]
P15998	ATP synthase, alpha subunit	Constant under anaerobic conditions	WB	Mitochondria	*O. sativa*/Howell *et al.*, 2007 [3]
Os09g08910	ATP synthase, alpha subunit	Decreased	iTRAQ	Whole seed	*O. sativa*/Han *et al.*, 2014 [1]
CBY80071	ATP synthase beta subunit	Decreased	2DE	Whole seed	*P. sativum*/Wang *et al.*, 2012 [4]
P19023	ATP synthase beta subunit	Constant	WB	Mitochondria	*Z. mays*/Logan *et al.*, 2001 [10]
At5g08690	ATP synthase beta subunit	Constant	WB	Whole seed	*A. thaliana*/Law *et al.*, 2012 [5]
At5g08670	ATP synthase beta subunit	Increased and neosynthesized	2DE	Whole seed	*A. thaliana*/Galland *et al.*, 2014 [6]
Os01g49190	ATP synthase, beta subunit	Increased under aerobic conditions	2DE	Mitochondria	*O. sativa*/Howell *et al.*, 2006 [2]
		**Tricarboxylic acid cycle/Carbon metabolism**			
P31023	Dihydrolipoyl dehydrogenase, mitochondrial	Increased	2DE	Whole seed	*P. sativum*/Wang *et al.*, 2012 [4]
At3g17240 or At1g48030	Dihydrolipoamide dehydrogenase 2 or 1 mitochondrial	Increased and NOT neosynthesized	2DE	Whole seed	*A. thaliana*/Galland *et al.*, 2014 [6]
B4FML9	Pyruvate dehydrogenase E1, alpha subunit	Increased	WB	Mitochondria	*Z. mays*/Logan *et al.*, 2001 [10]
Os02g0739600	Pyruvate dehydrogenase E1, alpha subunit	Increased under aerobic conditions	WB	Mitochondria	*O. sativa*/Howell *et al.*, 2006 [2]; *O. sativa*/Howell *et al.*, 2007 [3]
Os02g0739600	Pyruvate dehydrogenase E1, alpha subunit	Low and constant under anaerobic conditions	WB	Mitochondria	*O. sativa*/Howell *et al.*, 2007 [3]
AFJ15126	Pyruvate dehydrogenase E1, alpha subunit	Constant	2DE	Whole seed	*L. sativa*/Wang *et al.*, 2015 [9]

Atg59900	Pyruvate dehydrogenase E1, alpha subunit	Increased	2DE	Whole seed	*A. thaliana*/Fu *et al.*, 2005 [7]
Os09g33500	Putative pyruvate dehydrogenase E1, beta subunit, isoform 3	Increased under aerobic conditions	2DE	Mitochondria	*O. sativa*/Howell *et al.*, 2006 [2]
Os09g33500	Putative pyruvate dehydrogenase E1, beta subunit, isoform 1	Decreased under anaerobic conditions	2DE	Mitochondria	*O. sativa*/Howell *et al.*, 2007 [3]
Os06g30460/Os02g31040	Pyruvate dehydrogenase/2-oxo-glutarate dehydrogenase complex, E2 component	Increased under aerobic conditions	WB	Mitochondria	*O. sativa*/Howell *et al.*, 2006 [2]; *O. sativa*/Howell *et al.*, 2007 [3]
Os06g30460/Os02g31040	Pyruvate dehydrogenase/2-oxo-glutarate dehydrogenase complex, E2 component	Low and constant under anaerobic conditions	WB	Mitochondria	*O. sativa*/Howell *et al.*, 2007 [3]
B7ZWY9	Citrate synthase	Increased	WB	Mitochondria	*Z. mays*/Logan *et al.*, 2001 [10]
At2g44350	Citrate synthase	Increased and NOT neosynthesized	2DE	Whole seed	*A. thaliana*/Galland *et al.*, 2014 [6]
At2g05710	Citrate hydro-lyase/aconitase, putative	Increased	MS	Whole seed	*A. thaliana*/Law *et al.*, 2012 [5]
At2g05710	Aconitate hydratase 3	Increased and neosynthesized	2DE	Whole seed	*A. thaliana*/Galland *et al.*, 2014 [6]
At5g08300	Succinyl-CoA ligase (GDP-forming) alpha-chain, mitochondrial	Constant and neosynthesized	2DE	Whole seed	*A. thaliana*/Galland *et al.*, 2014 [6]
At2g20420	Succinyl-CoA ligase beta-chain (mitochondrial precursor)	Constant and NOT neosynthesized	2DE	Whole seed	*A. thaliana*/Galland *et al.*, 2014 [6]
Os02g40830	Putative succinyl-CoA ligase (GDP-forming) beta-chain, mitochondrial precursor	Increased under aerobic conditions	2DE	Mitochondria	*O. sativa*/Howell *et al.*, 2006 [2]
Os02g40830	Putative succinyl-CoA ligase (GDP-forming) beta-chain, mitochondrial precursor	Decreased under anaerobic conditions	2DE	Mitochondria	*O. sativa*/Howell *et al.*, 2007 [3]
At5g66760	Succinate dehydrogenase [ubiquinone] flavoprotein subunit, mitochondrial	Constant and neosynthesized	2DE	Whole seed	*A. thaliana*/Galland *et al.*, 2014 [6]

Os07g04240	Succinate dehydrogenase [ubiquinone] flavoprotein subunit, mitochondrial	Decreased	2DE	Whole seed	*O. sativa*/Han *et al.*, 2014 [1]
At1g53240	Malate dehydrogenase (NAD), mitochondrial	Increased	MS	Whole seed	*A. thaliana*/Law *et al.*, 2012 [5]
At3g47520	Malate dehydrogenase	Increased	MS	Whole seed	*A. thaliana*/Law *et al.*, 2012 [5]
At1g53240	Malate dehydrogenase (mitochondrial precursor)	Increased and NOT neosynthesized	2DE	Whole seed	*A. thaliana*/Galland *et al.*, 2014 [6]
At1g53240	Malate dehydrogenase (NAD), mitochondrial	Increased	2DE	Whole seed	*A. thaliana*/Fu *et al.*, 2005 [7]
Os01g46070	Malate dehydrogenase, NAD-dependent	Increased under aerobic conditions	2DE	Mitochondria	*O. sativa*/Howell *et al.*, 2006 [2]
At4g37870	Phosphoenolpyruvate carboxykinase (ATP)	Increased	MS	Whole seed	*A. thaliana*/Law *et al.*, 2012 [5]
At4g37870	Phosphoenolpyruvate carboxykinase (ATP)	Increased	2DE	Whole seed	*A. thaliana*/Gallardo *et al.*, 2001 [11]
At4g37870	Phosphoenolpyruvate carboxykinase (ATP)	Increased and neosynthesized	2DE	Whole seed	*A. thaliana*/Galland *et al.*, 2014 [6]
At4g37870	Phosphoenolpyruvate carboxykinase (ATP)	Increased	2DE	Whole seed	*A. thaliana*/Fu *et al.*, 2005 [7]
At5g14780	Formate dehydrogenase (FDH)	Constant and NOT neosynthesized	2DE	Whole seed	*A. thaliana*/Galland *et al.*, 2014 [6]
		**Import/Transport**			
Q5JJI4	Tom20	Decreased under aerobic conditions	WB	Mitochondria	*O. sativa*/Howell *et al.*, 2006 [2]; *O. sativa*/Howell *et al.*, 2007 [3]
Q5JJI4	Tom20	High and decreased under anaerobic conditions	WB	Mitochondria	*O. sativa*/Howell *et al.*, 2007 [3]
At3g20000	Tom40	Increased	WB	Whole seed	*A. thaliana*/Law *et al.*, 2012 [5]

Os01g16910	Tom40	Decreased under aerobic conditions	2DE	Mitochondria	*O. sativa*/Howell *et al.*, 2006 [2]
Os03g19290	Import inner membrane translocase subunit (TIM17/TIM22/TIM23 family protein)	Decreased under aerobic conditions	2DE	Mitochondria	*O. sativa*/Howell *et al.*, 2006 [2]
Os03g19290	Import inner membrane translocase subunit (TIM17/TIM22/TIM23 family protein)	Increased under anaerobic conditions	2DE	Mitochondria	*O. sativa*/Howell *et al.*, 2007 [3]
Os01g19770	Stress-inducible membrane pore protein (TIM17/TIM22/TIM23 family protein)	Decreased under aerobic conditions	2DE	Mitochondria	*O. sativa*/Howell *et al.*, 2006 [2]
Os01g19770	Stress-inducible membrane pore protein (TIM17/TIM22/TIM23 family protein)	Increased under anaerobic conditions	2DE	Mitochondria	*O. sativa*/Howell *et al.*, 2007 [3]
At1g20350	Tim17-1	Constant	WB	Whole seed	*A. thaliana*/Law *et al.*, 2012 [5]
At2g37410	Tim17-2	Increased	WB	Whole seed	*A. thaliana*/Law *et al.*, 2012 [5]
At4g16160	Mitochondrial import inner membrane translocase subunit	Decreased	2DE	Whole seed	*A. thaliana*/Fu *et al.*, 2005 [7]
Os09g19734	Mitochondrial outer membrane protein, porin	Increased	iTRAQ	Whole seed	*O. sativa*/Han *et al.*, 2014 [1]
Os05g45950	Outer mitochondrial membrane porin	Decreased	iTRAQ	Whole seed	*O. sativa*/Han *et al.*, 2014 [1]
Os09g19734	VDAC	Constant under aerobic conditions or increased under aerobic conditions	WB	Mitochondria	*O. sativa*/Howell *et al.*, 2006 [2]; *O. sativa*/Howell *et al.*, 2007 [3]
Os09g19734	VDAC	Increased under anaerobic conditions	WB	Mitochondria	*O. sativa*/Howell *et al.*, 2007 [3]
Q9SPD9, Q9SPD8, Q9SPD7	Porin	Increased	WB	Mitochondria	*Z. mays*/Logan *et al.*, 2001 [10]
At3g01280, At5g67500, At5g15090, At5g57490, At3g49920	VDAC	Increased	WB	Whole seed	*A. thaliana*/Law *et al.*, 2012 [5]

At5g15090	VDAC3	Increased	2DE	Whole seed	*A. thaliana*/Fu *et al.*, 2005 [7]
Os02g48720/Os05g23720	Adenine nucleotide translocator	Slightly decreased under anaerobic and aerobic conditions	MS	Mitochondria	*O. sativa*/Taylor *et al.*, 2010 [8]
P04709, P12857	Adenine nucleotide translocator	Increased	WB	Mitochondria	*Z. mays*/Logan *et al.*, 2001 [10]
At3g08580	AAC1 (ADP/ATP CARRIER 1); ATP:ADP antiporter/ binding	Increased	MS	Whole seed	*A. thaliana*/Law *et al.*, 2012 [5]
At5g13490	AAC2 (ADP/ATP CARRIER 2); ATP:ADP antiporter/ binding	Increased	MS	Whole seed	*A. thaliana*/Law *et al.*, 2012 [5]
Os02g52860, Os06g10810	Mitochondrial phosphate transporter	Constant under anaerobic and decreased under aerobic conditions	MS	Mitochondria	*O. sativa*/Taylor *et al.*, 2010 [8]
Os05g11780	Dicarboxylate/tricarboxylate carrier	Slightly increased under anaerobic and aerobic conditions	MS	Mitochondria	*O. sativa*/Taylor *et al.*, 2010 [8]
Os11g48040	Putative uncoupling protein	Increased under anaerobic and decreased under aerobic conditions	MS	Mitochondria	*O. sativa*/Taylor *et al.*, 2010 [8]
Os11g48040	Uncoupling protein	Increased under aerobic conditions	WB	Mitochondria	*O. sativa*/Howell *et al.*, 2006 [2]
Os10g42299	Basic amino acid carrier	Increased under anaerobic and aerobic conditions	SRM	Mitochondria	*O. sativa*/Taylor *et al.*, 2010 [8]
Os02g10800	Mitochondrial carrier protein	Decreased	iTRAQ	Whole seed	*O. sativa*/Han *et al.*, 2014 [1]
Os02g48720	Mitochondrial carrier protein	Decreased	iTRAQ	Whole seed	*O. sativa*/Han *et al.*, 2014 [1]
		**Stress response**			
Os05g25850	Superoxide dismutase	Increased	2DE	Whole seed	*O. sativa*/Han *et al.*, 2014 [1]
Os05g25850	Manganese superoxide dismutase	Decreased under aerobic	2DE	Mitochondria	*O. sativa*/Howell *et al.*,

		conditions			2006 [2]
P27084	Superoxide dismutase [Mn], mitochondrial	Increased	2DE	Whole seed	*P. sativum*/Wang *et al.*, 2012 [4]
ABH11434	Mn-superoxide dismutase II	Increased	2DE	Whole seed	*L. sativa*/Wang *et al.*, 2015 [9]
At3g56350	Superoxide dismutase (Mn), MSD2	Decreased and neosynthesized	2DE	Whole seed	*A. thaliana*/Galland *et al.*, 2014 [6]
At3g10920	Superoxide dismutase (Mn), MSD1	Decreased and NOT neosynthesized	2DE	Whole seed	*A. thaliana*/Galland *et al.*, 2014 [6]
Os01g58970	Cytochrome P450 protein	Increased	2DE	Whole seed	*O. sativa*/Han *et al.*, 2014 [1]
At1g20620	Catalase 3	Increased	MS	Whole seed	*A. thaliana*/Law *et al.*, 2012 [5]
At1g20620	Catalase 3	Increased	2DE	Whole seed	*A. thaliana*/Fu *et al.*, 2005 [7]
		**Chaperones and proteolytic system**			
Os01g39250	OsFTSH4 FTSH protease, homologue of AtFTSH4	Increased	iTRAQ	Whole seed	*O. sativa*/Han *et al.*, 2014 [1]
Os02g57060	C-terminal processing peptidase homologue	Increased	2DE	Whole seed	*O. sativa*/Han *et al.*, 2014 [1]
XP_002284370	Probable mitochondrial-processing peptidase subunit beta	Decreased	2DE	Whole seed	*L. sativa*/Wang *et al.*, 2015 [9]
At3g02090	Putative mitochondrial processing peptidase subunit beta	Increased and neosynthesized	2DE	Whole seed	*A. thaliana*/Galland *et al.*, 2014 [6]
Os01g53700, Os0g09560	Mitochondrial processing peptidase subunit alpha	Decreased under anaerobic conditions	2DE	Mitochondria	*O. sativa*/Howell *et al.*, 2007 [3]
Os03g11410	Mitochondrial processing peptidase subunit beta	Decreased under anaerobic conditions	2DE	Mitochondria	*O. sativa*/Howell *et al.*, 2007 [3]
At5g20720	Chaperonin 20; calmodulin binding	Increased	MS	Whole seed	*A. thaliana*/Law *et al.*, 2012 [5]
Os12g32990	Putative heat-shock protein	Increased under aerobic conditions	2DE	Mitochondria	*O. sativa*/Howell *et al.*, 2006 [2]
Os10g32550	T-complex protein/Chaperonin CPN60-1,	Increased	iTRAQ	Whole seed	*O. sativa*/Han *et al.*, 2014

	mitochondrial				[1]
Os10g32550	Mitochondrial chaperonin-60 HSP60	Increased under aerobic conditions or constant under aerobic conditions	2DE, WB	Mitochondria	*O. sativa*/Howell *et al.*, 2006 [2]
Os10g32550	Mitochondrial chaperonin-60 HSP60	Increased, then decreased	2DE	Whole seed	*O. sativa*/Han al., 2014 [1]
Os10g32550	Mitochondrial chaperonin-60 HSP60	Constant under anaerobic conditions	WB	Mitochondria	*O. sativa*/Howell *et al.*, 2007 [3]
P29185, Q43298	Chaperonin CPN60-2, putative	Increased	WB	Mitochondria	*Z. mays*/Logan *et al.*, 2001 [10]
At3g13470	Chaperonin CPN60, mitochondrial	Increased	MS	Whole seed	*A. thaliana*/Law *et al.*, 2012 [5]
At3g23990	HSP60	Increased and neosynthesized	2DE	Whole seed	*A. thaliana*/Galland *et al.*, 2014 [6]
At2g33210	Chaperonin CPN60-2, mitochondrial	Increased and neosynthesized	2DE	Whole seed	*A. thaliana*/Galland *et al.*, 2014 [6]
P11143	HSP70	Increased	WB	Mitochondria	*Z. mays*/Logan *et al.*, 2001 [10]
At3g12580	HSP70	Increased	MS	Whole seed	*A. thaliana*/Law *et al.*, 2012 [5]
At5g09590	HSP70-2	Constant and neosynthesized	2DE	Whole seed	*A. thaliana*/Galland *et al.*, 2014 [6]
Os03g02260	Putative heat shock 70 kDa protein, mitochondrial precursor	Increased under aerobic conditions	2DE, WB	Mitochondria	*O. sativa*/Howell *et al.*, 2006 [2]; *O. sativa*/Howell *et al.*, 2007 [3]
Os02g53420	Putative heat shock 70 kDa protein, mitochondrial precursor	Increased under aerobic conditions	2DE, WB	Mitochondria	*O. sativa*/Howell *et al.*, 2006 [2]
Os03g02260	Putative heat shock 70 kDa protein, mitochondrial precursor	Constant or decreased under anaerobic conditions	2DE, WB	Mitochondria	*O. sativa*/Howell *et al.*, 2007 [3]
		**Development**			
Os04g52110	Late embryogenesis abundant protein,	Increased	iTRAQ	Whole seed	*O. sativa*/Han *et al.*, 2014

	group 3, putative				[1]
Os04g52110	Late embryogenesis abundant protein, group 3, putative	Increased under anaerobic conditions	2DE	Mitochondria	*O. sativa*/Howell *et al.*, 2007 [3]
Os01g50910	Late embryogenesis abundant protein, group 3	Decreased	iTRAQ	Whole seed	*O. sativa*/Han *et al.*, 2014 [1]
Os03g20680	Late embryogenesis abundant protein	Decreased under aerobic conditions/Decreased	2DE/iTRAQ	Mitochondria/ Whole seed	*O. sativa*/Howell *et al.*, 2006 [2];*O. sativa*/Han *et al.*, 2014 [1]
At4g21020	Late embryogenesis abundant protein (LEA)	Decreased and NOT neosynthesized	2DE	Whole seed	*A. thaliana*/Galland *et al.*, 2014 [6]
		**Protein synthesis**			
At1g26880	60S ribosomal protein L34 (RPL34A)	Increased	MS	Whole seed	*A. thaliana*/Law *et al.*, 2012 [5]
At4g02930	Translation elongation factor EF-Tu precursor, mitochondrial	Increased and neosynthesized	2DE	Whole seed	*A. thaliana*/Galland *et al.*, 2014 [6]
Os03g63410	Translational elongation factor Tu	Increased under aerobic conditions	2DE	Mitochondria	*O. sativa*/Howell *et al.*, 2006 [2]
Os03g63410	Translational elongation factor Tu	Decreased under anaerobic conditions	2DE	Mitochondria	*O. sativa*/Howell *et al.*, 2007 [3]
At2g45030	Mitochondrial elongation factor	Increased	2DE	Whole seed	*A. thaliana*/Fu *et al.*, 2005 [7]
		**Replication**			
At5g59440	Thymidylate kinase family protein	Increased	MS	Whole seed	*A. thaliana*/Law *et al.*, 2012 [5]
		**RNA binding**			
At5g46460	Pentatricopeptide (PPR) repeat-containing protein	Decreased	MS	Whole seed	*A. thaliana*/Law *et al.*, 2012 [5]
		**Redox homeostasis**			
At5g42980	Thioredoxin H-type 3; thiol-disulfide exchange intermediate	Decreased	MS	Whole seed	*A. thaliana*/Law *et al.*, 2012 [5]
		**Unknown function**			
At5g54770	THI1, thiazole recquiring	Increased	MS	Whole seed	*A. thaliana*/Law *et al.*, 2012 [5]

2DE: Two-Dimensional Gel Electrophoresis; iTRAQ: Isobaric Tags for Relative and Absolute Quantitation; MS: Mass Spectrometry; SRM: Selected Reaction Monitoring; WB: Western Blotting

Table S2. Overview of carbonylated, phosphorylated and S-nitrosylated mitochondrial proteins identified in germinating seeds using gel-based and gel-free proteomic approaches in different plant species.

Carbonylated Mitochondrial Proteins in Seeds					
**Accession Number (UniProt)**	**Name of Protein**	**Change in Carbonylation during Germination**	**Method**	**Studied Sample**	**Species/References**
**Stress response/Redox homeostasis**					
B7ERQ1	Peroxiredoxin	Significantly increased in carbonylation level	MS	Whole seed	*O. sativa*/Zhang *et al.*, 2016 [12]
Q43803	Superoxide dismutase, Mn	*Not determined*	MS	Whole seed	*O. sativa*/Zhang *et al.*, 2016 [12]
**Chaperones**					
Q8GTB0	Putative heat shock 70 KD protein, mitochondrial	Significantly increased in carbonylation level	MS	Whole seed	*O. sativa*/Zhang *et al.*, 2016 [12]
Q9LDZ0	Heat shock protein Hsp70-10	Increased during germination	2DE	Whole seed	*A. thaliana*/Job *et al.*, 2005 [13]
Q10RW9	Chaperonin, probable CPN60-1	Gradually decreasing in carbonylation level	MS	Whole seed	*O. sativa*/Zhang *et al.*, 2016 [12]
**Metabolism**					
Q93Y73	Aspartate-semialdehyde dehydrogenase family protein, mitoch./chloropl.	Gradually increasing in carbonylation level	MS	Whole seed	*O. sativa*/Zhang *et al.*, 2016 [12]
Q2QMG2	Methylcrotonoyl-CoA carboxylase subunit alpha, mitochondrial	Gradually decreasing in carbonylation level	MS	Whole seed	*O. sativa*/Zhang *et al.*, 2016 [12]
**Import**					
Q0DYM2	Zinc finger, Tim10/DDP-type family protein	*Not determined*	MS	Whole seed	*O. sativa*/Zhang *et al.*, 2016 [12]
**Mitochondrial trafficking**					
Q6ATR5	Mitochondrial Rho GTPase	*Not determined*	MS	Whole seed	*O. sativa*/Zhang *et al.*, 2016 [12]
**Tricarboxylic acid cycle**					

Q9SIB9	Aconitate hydratase (ACO2)	Increased during germination	2DE	Whole seed	*A. thaliana*/Job *et al.*, 2005 [13]
Q9XGU8	Isocitrate dehydrogenase [NADP]	Gradually increasing in carbonylation level	MS	Whole seed	*O. sativa*/Zhang *et al.*, 2016 [12]
Q7XMA0	Isocitrate dehydrogenase [NADP]	Significantly increased in carbonylation level	MS	Whole seed	*O. sativa*/Zhang *et al.*, 2016 [12]
B9FVD6	Putative uncharacterized protein, probable SDH1	*Not determined*	MS	Whole seed	*O. sativa*/Zhang *et al.*, 2016 [12]
		**Respiration**			
Q0JKB4	ATP synthase subunit beta, mitochondrial	Gradually decreasing in carbonylation level	MS	Whole seed	*O. sativa*/Zhang *et al.*, 2016 [12]
P83483, Q9C5A9, P83484	ATP synthase subunit beta, mitochondrial precursor	Increased during germination	2DE	Whole seed	*A. thaliana*/Job *et al.*, 2005 [13]
		**Unknown function**			
A3AXT8	Putative uncharacterized protein	Gradually decreasing in carbonylation level	MS	Whole seed	*O. sativa*/Zhang *et al.*, 2016 [12]
Q6AT24	Os05g0176500 protein	*Not determined*	MS	Whole seed	*O. sativa*/Zhang *et al.*, 2016 [12]
Q7Y0E6	LOC496049 protein, putative	*Not determined*	MS	Whole seed	*O. sativa*/Zhang *et al.*, 2016 [12]
		Phosphorylated mitochondrial proteins in seeds			
		**Chaperones**			
O64960	Heat shock protein HSP22	*Not determined*	MS	Whole seed	*Z. mays*/Lu *et al.*, 2008 [14]
		**Tricarboxylic acid cycle**			
Q6Z5N4	Similar to pyruvate dehydrogenase E1 component alpha subunit-mitochondrial precursor (PDHE1-A)	*Not determined*	MS	Whole seed	*O. sativa*/Han *et al.*, 2014 [15]
		**Respiration**			
Q1KKK0	Cytochrome *c* biogenesis FN	*Not determined*	MS	Whole seed	*Z. mays*/Lu *et al.*, 2008 [14]
Q8GT70	Alternative oxidase AOX3 precursor	*Not determined*	MS	Whole seed	*Z. mays*/Lu *et al.*, 2008

					[14]
		**Protein synthesis**			
B7SDE4	60S ribosomal protein L5	Increased	MS	Whole seed/Nucleus	*O. sativa*/Li *et al.*, 2015 [16]
A0A0P0VB K7	Ribosomal protein L18/L5 domain containing protein	Increased	MS	Whole seed/Nucleus	*O. sativa*/Li *et al.*, 2015 [16]
		S-nitrosylated mitochondrial proteins in seeds			
P83483, Q9C5A9, P83484	ATP synthase subunit beta	*Not determined*	1DE	Whole seed	*A. thaliana*/Arc *et al.*, 2011 [17]

1DE: One-Dimensional Gel Electrophoresis; 2DE: Two-Dimensional Gel Electrophoresis; MS: Mass Spectrometry.

References

1. Han, Z.; He, D.; Li, M.; Yang, P. In-depth proteomic analysis of rice embryo reveals its important roles in seed germination. *Plant Cell Physiol.* **2014**, *55*, 1826–1847.
2. Howell, K.A.; Millar, A.H.; Whelan, J. Ordered assembly of mitochondria during rice germination begins with promitochondrial structures rich in component of the protein import apparatus. *Plant Mol. Biol*. **2006**, *60*, 201–223.
3. Howell, K.A.; Cheng, K.; Murcha, M.W.; Jenkin, L.E.; Millar, A.H.; Whelan, J. Oxygen initiation of respiration and mitochondrial biogenesis in rice. *J. Biol. Chem*. **2007**, *282*, 15619–15631.
4. Wang, W.-Q.; Møller, I.M.; Song, S.-Q. Proteomic analysis of embryonic axis of Pisum sativum seeds during germination and identification of proteins associated with loss of desiccation tolerance. *J. Proteom.* **2012**, *77*, 68–86.
5. Law, S.R.; Narsai, R.; Taylor, N.L.; Delannoy, E.; Carrie, C.; Giraud, E.; Millar, A.H.; Small, I.; Whelan, J. Nucleotide and RNA metabolism prime translational initiation in the earliest events of mitochondrial biogenesis during Arabidopsis germination. *Plant Physiol*. **2012**, *158*, 1610–1627.
6. Galland, M.; Huguet, R.; Aec, E.; Cueff, G.; Job, D.; Rajjou, L. Dynamic proteomics emphasizes the importance of selective mRNA translation and protein turnover during Arabidopsis seed germination. *Mol. Cell Proteom.* **2014**, *13*, 252–268.
7. Fu, Q.; Wang, B.-C.; Jin, X.; Li, H.-B.; Han, P.; Wei, K.-H.; Zhang, X.-M.; Zhu, Y.-X. Proteomic analysis and extensive protein identification from dry, germinating Arabidopsis seed and young seedlings. *J. Biochem. Mol. Biol*. **2005**, *38*, 650–660.
8. Taylor, N.L.; Howell, K.A.; Heazlewood, J.L.; Tan, T.Y.W.; Narsai, R.; Huang, S.; Whelan, J.; Millar, A.H. Analysis of the rice mitochondrial carrier family reveals anaerobic accumulation of a basic amino acid carrier involved in arginine metabolism during seed germination. *Plant Physiol*. **2010**, *154*, 691–704.
9. Wang, W.-Q.; Song, B.-Y.; Deng, Z.-Y.; Wang, Y.; Liu, S.-J.; Møller, I.M.; Song, S.-Q. Proteomic analysis of lettuce seed germination and thermoinhibition by sampling of individual seeds at germination and removal of storage proteins by polyethylene glycol fractionation. *Plant Physiol*. **2015**, *167*, 1332–1350.
10. Logan, D.C.; Millar, A.H.; Sweetlove, L.J.; Hill, S.A.; Leaver, C.J. Mitochondrial biogenesis during germination in maize embryos. *Plant Physiol*. **2001**, *125*, 662–672.
11. Gallardo, K.; Job, C.; Groot, S.P.C.; Puype, M.; Demol, H.; Vandekerckhove, J.; Job, D. Proteomic analysis of Arabidopsis seed germination and priming. *Plant Physiol*. **2001**, *126*, 835–848.
12. Zhang, H.; He, D.; Yu, J.; Li, M.; Damaris, R.N.; Gupta, R.; Kim, S.T.; Yang, P. Analysis of dynamic protein carbonylation in rice embryo during germination through AP-SWATH. *Proteomics* **2016**, *16*, 989–1000, doi:10.1002/pmic.201500248.
13. Job, C.; Rajjou, L.; Lovigny, Y.; Belghazi, M.; Job, D. Patterns of protein oxidation in arabidopsis seeds and during germination. *Plant Physiol.* **2005**, *138*, 790–802.
14. Lu, C.-T.; Meng, L.-B.; Yang, C.-P.; Liu, G.-F.; Liu, G.-J.; Ma, W.; Wang, B.-C. A shotgun phosphoproteomics analysis of embryos in germinated maize seeds. *Planta* **2008**, *228*, 1029–1041.
15. Han, C.; Yang, P.; Sakata, K.; Komatsu, S. Quantitative Proteomics Reveals the Role of Protein Phosphorylation in Rice Embryos during Early Stages of Germination. *J. Proteome Res*. **2014**, *13*, 1766–1782.
16. Li, M.; Yin, X.; Sakata, K.; Yang, P.; Komatsu, S. Proteomic analysis of phosphoproteins in the rice nucleus during the early stage of seed germination. *J. Proteome Res*. **2015**, *14*, 2884–2896.
17. Arc, E.; Galland, M.; Cueff, G.; Godin, B.; Lounifi, I.; Job, D.; Rajjou, L. Reboot the system thanks to protein post-translational modifications and proteome diversity: How quiescent seeds restart their metabolism to prepare seedling establishment. *Proteomics* **2011**, *11*, 1606–1618.

© 2016 by the authors. Submitted for possible open access publication under the terms and conditions of the Creative Commons Attribution (CC-BY) license (http://creativecommons.org/licenses/by/4.0/).
